# A cross-sectional study of mother-child agreement on PTSD symptoms in a south Indian post-tsunami sample

**DOI:** 10.1186/s12888-019-2408-9

**Published:** 2019-12-21

**Authors:** Silvia Exenberger, David Riedl, Kumuthavalli Rangaramanujam, Vijai Amirtharaj, Florian Juen

**Affiliations:** 1 Department of Medical Psychology, Medical University of Innsbruck, Speckbacherstr, 23/3, 6020 Innsbruck, Austria; 2 Department of Social Work, Pondicherry University, Pondicherry, 605014 India; 3 Department of Psychology, University of Innsbruck, Bruno-Sander-Haus, Innrain 52f, 6020 Innsbruck, Austria

**Keywords:** Posttraumatic stress disorder, Caregiver-child agreement, India, Natural disaster

## Abstract

**Background:**

Few studies examine caregiver-child agreement on posttraumatic stress disorder (PTSD) symptoms in non-Western cultures. The present study investigated mother-child agreement for PTSD symptoms in a South Indian sample, which was affected by the Indian Ocean Tsunami in 2004.

**Methods:**

Data was collected four years post-disaster. In total, 80 mothers rated PTSD symptoms for their 164 children and gave information about their own trauma symptoms. In addition, the children aged 8 to 17 reported about their own PTSD symptoms.

**Results:**

Results showed that mother-child agreement on posttraumatic stress symptoms was poor, and a child’s age, gender and living situation (fishing village vs. family-based out-of-home care) did not positively influence this concordance. Moreover, mothers’ own posttraumatic symptoms were strongly related to maternal reports of the child’s PTSD symptoms. Multivariate analyses showed that mothers’ PTSD symptoms were the only significant predictor for discrepancies in the rating of the child’s PTSD symptoms. That means, if mothers reported clinically relevant PTSD symptoms, the likelihood for disagreement on the child’s PTSD ratings more than doubled. Neither age, nor gender nor the living situation had an influence on children’s self-rated posttraumatic stress reactions.

**Conclusions:**

In general, long-term monitoring of posttraumatic stress symptoms of mothers and children should be planned by relief actions as recovery processes are decelerated through lacking resources in developing countries such as India. Specifically, the assessment of mothers’ trauma symptoms is inevitable because the mothers’ own responses to disaster highly influence their assessment of their children’s symptoms. Mother-child agreement is discussed against the background of socio-cultural aspects.

## Background

It is a commonly accepted practice among experts to obtain information from multiple informants to generate a comprehensive picture regarding childhood disorders [1, 2]. The relationship between caregiver and child reports of child psychopathology is well studied with regard to other anxiety disorders [3] and child problem behaviours [4, 5], whereas the literature on this topic is scarce for PTSD [6]. Studies that addressed parent-child agreement in relation to diverse childhood disorders were mainly implemented in Western settings [7]. In cases where interrater agreement was investigated in non-Western cultures, child-parent dyads belonged to minority groups living in the United States [7, 8]. The present study investigated symptom agreement for PTSD according to DSM-IV criteria between Tamil children and their mothers in the long-term aftermath of the Indian Ocean Tsunami. At this point it has to be noted that the diagnostic algorithms used in the studies of the following review of the research literature concerning the concordance between parent and child reports on diverse childhood disorders conformed to DSM-IV [9] criteria.

In general, caregivers and children rarely reported overlapping information whenever they independently assessed the presence of childhood disorders [10, 11]. Specifically, caregiver-child agreement was poor with regard to other anxiety disorders [12, 13], and low to modest regarding child problem behaviour [4, 5]. These findings indicate that the extent of agreement between child and parent reports is higher on externalising symptoms and lower on internalising ones [14], because internalising symptoms are of a very subjective nature and directly accessible only to children [15]. With regard to PTSD, several scholars measured parent-child agreement for interview-based diagnoses of Acute Stress Disorder (ASD) [6] and PTSD [6, 16] in children and found that caregivers were under-reporting ASD [6] and PTSD [6, 16] symptoms in their children relative to child reports, whereas children were over-reporting symptoms relative to parent reports. However, Meiser-Stedman et al. [6] and Schreier et al. [17] ascertained a decrease of the discrepancy between the reports of caregivers and children for the child PTSD symptom clusters intrusion, avoidance and hyper-arousal over time. While the authors [6, 17] found higher caregiver-child agreement for the hyper-arousal cluster compared to the re-experience and avoidance criterions, other researchers [2, 18] reported high caregiver-child agreement for the re-experiencing cluster and low agreement for the avoidance and hyper-arousal cluster. The overall results seem inhomogeneous.

The most investigated factors that may have an impact on parent-child agreement were children’s age and gender and the caregiver’s own psychopathology. Regarding age, some studies on anxiety disorders showed better agreement between older children and their parents [11, 19], whereas other scholars observed this tendency between younger children and parents [20]. However, the majority of studies found no significant influence of age on the agreement of child-caregiver reports for anxiety disorders [12, 13, 21] and posttraumatic stress symptoms (PTS) [22]. With regard to PTSD symptoms, Semesh et al. [24] found that the gap between adolescents’ (above 12 years of age) reports of their symptoms and that of their parents was greater than that of children under the age of 12. Stover et al. [18] found high concordance between the reports of school-aged children and those of their parents concerning re-experiencing symptoms on the one hand and low agreement concerning avoidance symptoms on the other. The same was found for adolescents and their parents, but adolescents and their parents additionally agreed on the presence of hyper-arousal symptoms [18]. To our knowledge there is no study, except for Stover et al.’s [18], that assessed the child-parent concordance for PTSD by gender, indicating a significantly higher agreement between boys and parents than between girls and parents for the hyper-arousal cluster. In general, the effect of gender on parent-child agreement is reported as insignificant [12, 21, 23]. With regard to the influence of age and gender on children’s self-reported PTSD symptoms findings are inhomogeneous. Concerning child self-reported trauma symptoms, a study conducted one year after the 2004 Tsunami in India found that girls generally reported more trauma symptoms than boys [25]. In contrast, male and female Malaysian adolescents reported equal PTSD symptoms four years after the 2004 Tsunami disaster in the study of Ghazali et al. [26], and a similar result was found for Sri Lankan children soon after the Tsunami [27]. With regard to age, Vijayakumar et al. [28] detected no age differences (above and below the age of 12) in the scores on trauma symptoms in an Indian Tsunami-affected sample one year post-disaster. However, age and gender did not predict posttraumatic symptoms in Sri Lankan children four weeks after the 2004 Tsunami [29].

A strong finding regarding the effect of caregivers’ trauma symptoms on their reporting of symptoms in children is the association between poor psychological functioning in the caregiver and increased trauma symptoms in the child [30, 31]. Kassam-Adams et al. [32] investigated parent-child concordance for ASD symptoms in children aged 8–17, who were hospitalised for injuries after a street accident. The authors reported that parents with subsyndromal/full ASD on average rated their children’s ASD higher than the children rated themselves. In contrast, parents with no ASD rated their children’s ASD lower than the children rated themselves. These findings implied that all parents, whether symptomatic or not, were vulnerable to wrongly estimating their children’s symptoms in either direction [32]. Semesh et al. [24] found that in general, parents’ own distress was significantly correlated with parents’ reports of their children’s PTSD symptoms. The findings of Exenberger and Juen’s [33] qualitative study with Tsunami-affected mothers, who also were participants of the current quantitative study, substantiated the effect of caregivers’ trauma symptoms on their reporting of symptoms in children as mentioned by several authors [30–32]. Even though mothers were asked to describe their children’s well-being, they mainly portrayed their ill-being, which they characterised by trauma symptoms such as clinginess, sleep disturbances and jumpiness. In addition, mothers revealed a lot about their own ill-being [33]. As being mainly widows – a social status that put Indian women at risk [34] – mothers had to struggle with their economic and physical survival, thus, they perceived posttraumatic stress symptoms of their children as an additional burden to their burdensome life [33].

Research findings indicated that parent-child agreement regarding child’s symptomatology differ as a function of ethnicity. In the study of Weems et al. [23] Afro-American parents and children were less in agreement than European-Americans. The authors concluded that this discrepancy may be due to less emotion-related communication between parents and children because core values of the Afro-American culture are respect and deference to authority, i.e. these core values rather prohibit than encourage an emotion-related communication. Also van de Looij-Jansen et al. [7], who investigated parent-child concordance for internalising child problem behaviour in immigrant groups in the Netherlands, noticed that discrepancy scores differed significantly by ethnic background. Similar to Weems et al. [23], the authors attributed the reporters’ disagreement to different cultural values. In another study, Rousseau and Drapeau [8] compared the types and scores of psychiatric symptoms of Central American and Cambodian refugees as reported by both parents and children. The general profile of agreement in parent-child dyads of Central American and Cambodian origin differed from the profiles found in North-American population. More specifically, the Central American parent-child dyads agreed similarly on the intensity of internalising and externalising symptoms, whereas Combodians’ agreement for intensity of internalising symptoms was greater than for externalising symptoms. Thus, cultural influences on emotional problems need to be considered when assessing psychiatric symptoms in children belonging to different cultural backgrounds [8].

The purpose of the present study was to investigate symptom agreement for PTSD in a sample of Tsunami-affected children and their mothers living in Tamil Nadu, a South Indian state. The study explored the impact of a child’s age, gender and living situation (i.e. living in a fishing village vs. living in a family-based out-of-home care) as well as the caregiver’s own burden on this concordance. We hypothesised that (1) a child’s and mother’s reports on symptoms in the PTSD criterions intrusion, avoidance, and hyper-arousal do not significantly correlate, (2) a child’s own rating depends on his or her age, gender, and living situation, i.e. girls and older children score higher as well as children living with their parent(s) in a fishing village, (3) a mother’s rating of her children depends on her own symptomatology, i.e. symptomatic mothers report more PTSD symptoms in their children and (4) the mother-child discrepancies in the PTSD ratings are influenced by the child’s socio-demographic background (i.e. age, gender, living situation), and the mothers’ posttraumatic stress symptoms.

## Methods

### Setting

In the present study all children and the majority of their mothers faced the Indian Ocean Tsunami on December 26, 2004, which devastated the shorelines of several countries. In India, the Union Territories of Andaman, the Nicobar Islands and Puducherry, as well as the coastal areas of the South Indian states of Tamil Nadu, Kerala and Andhra Pradesh were most affected. The majority of those affected on the coast were fisher-folk [35]. All child participants and biological mothers lived in highly collectivistic surroundings, i.e. in small, single-caste fishing hamlets, that were isolated from the Tamil society and government [36]. This isolation originated from the villages’ political system, called “uur panchayat”, who possesses a large degree of power and represents both the individuals and the village as a whole to the external community. This practice ensures that fishermen tend to rally around their “uur panchayats” and work as a collective [36].

The children in this study belonged to two different groups (village children and SOS children) depending on their living arrangement. Children, who stayed with their Tsunami-affected parent(s) in severely damaged villages of the Nagapattinam district and at the Union Territory Puducherry, belonged to the group “village children”. The majority of the parents decided to stay in their original fishing hamlet due to their profession, i.e. fathers – depending on the severity of their injuries– working as fishermen and/or mothers working in the fishing business. They lived in thatched huts or self-constructed brick houses next to or near the sea. The remaining families moved to constructed settlements, adjacent to their old site, which were provided by the Government of Tamil Nadu or international NGOs, i.e. social networks were disrupted through relocation [33, 36]. Children, whose parents died due to the Tsunami, belonged to the group “SOS children”. They had to move to family-based out-of-home care, which was represented by SOS Children’s Villages, an international, independent non-governmental (NGO) and social development organisation [37]. The organisation focuses on children who lost parental care (e.g., Tsunami-affected children of the present study) and/or children who are at risk to lose it (e.g., abandoned children). A SOS village is equipped with clean water and electricity and provides education and health care for all children and SOS staff. We use the term mother for biological mothers and substitute mothers (SOS mothers). Originally, the group “SOS children” lived in fishing villages. Due to their loss of parents they had to move to SOS Children’s Villages. In India, a SOS mother is a substitute mother for about ten children. They were not affected by the Tsunami and lived with their SOS children in a stable house of a SOS village (10 to 15 family houses) away from the sea.

The first author of this study resided for two years in Puducherry and was co-operating with SOS Children’s Villages throughout the whole project. The overall sample was generally representative of the population living in South Indian (fishing) villages since they belonged to a similar caste and lived in villages with a similar structure (“uur panchayat” system).

### Participants

A total of 164 children participated in the study: 128 (78.0%) children belonged to the group “village children”, and 36 (22.0%) children belonged to the group “SOS children”. The complete sample comprised 71 boys (58 village boys, 13 SOS boys), and 93 girls (70 village girls, 23 SOS girls). Their ages ranged from 8 to 17 years with a mean age of 11.7 (*SD* = 2.6). The mean age of village and SOS children was 11.9 years (*SD* = 2.6) and 11.2 years (*SD* = 2.5) respectively. The children were divided into two age groups: “older girls/boys” (12 to 17 years), and “younger girls/boys” (8 to 11 years). In addition, 80 mothers of which 66 mothers (82.5%) were living with their children in the fishing villages (village mothers), and 14 SOS mothers (17.5%) gave information about their participating children and themselves.

Eligibility criteria were the children’s age (8 to 17 years), and direct exposure to the Tsunami. The children and their caregivers were of Hindu religion. The first author of this study recruited SOS mothers and SOS children via information sessions about the study at the respective SOS Children’s Villages. In case SOS mothers decided to participate, their Tsunami-affected children were approached. SOS co-workers recruited all village mothers and their children. They contacted the respective “uur panchayats” of the fishing hamlets and informed them about the study. The “uur panchayats” in turn approached the village mothers. The children were approached via their caregivers.

### Measures

#### Demographic characteristics

The children reported their age, gender and religion.

All scales used, are scored according to DSM-IV [9] criteria.

#### Children’s revised impact of event Scale-13 (CRIES-13), Tamil version [38]

This 13-item self-report screens children at risk for PTSD. It measures symptoms of intrusion, avoidance, and hyper-arousal according to the frequency of their occurrence related to the traumatic event (Tsunami) during the past week on a 4-point scale (0, 1, 3, 5; scale range: 0–65) with a cut-off score of 30 [39]. The CRIES-13 has satisfactory reliability for the total score (α = .80) and its subscales (intrusion: *α* = .70, avoidance: *α* = .73, arousal: *α* = .60) in a sample with war-affected Bosnian children [40].

#### Parent report of the child’s reaction to stress (PRCRS) [41]

The PRCRS is a 78-item parent report about the child’s reaction to exposure to a high-magnitude stressor (Tsunami). The first 51 questions allow DSM-IV PTSD criteria A-D to be assessed and diagnoses to be made. Additional 27 items assess associated symptoms since the occurrence of the event (e.g., anxiety, depression). Some of the items are followed by a request to explain or describe the reason for the response. Parents can estimate the symptoms of their children within the range from 4 to 19 years. Psychometrics are based on a small sample of 30 caregivers with acceptable levels of internal consistency for the total 51 PTSD items (α = .89) as well as for the criterion A items (α = .81), and the PTSD clusters of intrusion (α = .86), avoidance (α = .70) and hyper-arousal (α = .81) [41]. The Tamil version was developed using stringent back-translation procedures according to Weiss [42].

#### Impact of event scale-revised (IES-R) [43]

This scale is a self-report screening measure of current subjective distress in response to a specific traumatic event. Village mothers answered this scale with regard to the tsunami, and SOS mothers named their worst experience ever and answered the questions accordingly. The three subscales comprise the PTSD symptom clusters: intrusion, avoidance, and hyper-arousal. The IES-R is rated on how distressing each item has been during the past week on a 5-point scale (0, 1, 2, 3, 4; scale range: 0–88) with a cut-off score of 33 [44]. High levels of internal consistency have been reported for the total scale (α = .96) as well as for the three subscales intrusion (α = .94), avoidance (α = .87), and hyper-arousal (α = .91) with Vietnam veterans who joined a hospital-based PTSD treatment programme [37]. The Tamil version of the Impact of Event Scale was provided by Braj Bhushan, who investigated the psychological effects of the 2004 Tsunami on adolescents [25].

### Procedure

The present investigation was part of a large funded research project that was implemented four years post-disaster (data for the entire project were collected from February until October 2009). Two bilingual university students (male and female) were recruited for the entire research process. They not only acted as interpreters but also as cultural intermediaries. This includes the act of bridging or linking between groups or persons of different cultural backgrounds to facilitate collaboration.

Within the framework of the larger research project, oral and written informed consent were obtained from caregivers and children. That means for village children we obtained written and oral consent from their parents or mothers, and for SOS children we obtained written and oral consent from their legal guardians. A child-friendly version of an informed consent was handed out to the children. They gave their oral consent. At any stage of the research process children as well as caregivers could refuse further participation. The questionnaires were administered orally on an individual basis, and visual answering sheets were used. In case of psychological problems children could consult the psychologist or educationalist of SOS Children’s Villages, and caregivers could consult the first author of this study. Ethical permission to conduct the study was granted by the European Commission, who funded the entire research project.

### Data analysis

Descriptive statistics are given for socio-demographic and clinical data for children and their mothers. Associations between children’s and mother’s PTSD ratings were calculated using Pearson correlations. The influence of the children’s socio-demographic variables on their PTSD ratings were investigated by calculation of linear regressions. To evaluate whether the mother’s own symptomatology would influence their rating of the child’s symptoms, a linear regression analysis with the PRCRS value as dependent variable and the IES-R scores as independent variables was calculated. Finally, we calculated a logistic regression analysis to evaluate which factors influenced the mother-child agreement in the PTSD ratings. A dichotomous variable was calculated: agreement was given if the mother and child corresponded in their assessment of the absence or presence of clinically relevant PTSD symptoms; disagreement was given if the mother and child rated the absence or presence of clinically relevant PTSD symptoms differently. The children’s socio-demographic variables (age, gender, living situation) as well as the mothers PTSD symptoms (dichotomized as: clinically relevant PTSD vs. no clinically relevant PTSD) were entered as independent variables. Odds ratios (OR) and 95% Confidence Interval (95% CI) are reported. *P*-values < 0.05 were considered significant. Statistical analyses were performed with IBM SPSS statistics version 24.

## Results

On average, the included 164 children had a mean CRIES-13 total score of 19.3 points (*SD* = 10.2, range = 0–47), with 12.8% scoring above the proposed cut-off for PTSD. Mean scores for the intrusion subscale were 5.8 (*SD* = 4.6; range = 0–8), for avoidance 7.0 (*SD* = 5.2; range = 0–20), and for arousal 6.5 (*SD* = 4.6; range = 0–19). Mothers had a mean IES score of 24.1 (*SD* = 23.5; range = 0–75), with village mothers showing significantly higher values than SOS-mothers (27.9 vs. 14.3; *p* = .051). Overall, 31.3% of the mothers in the sample had scores above the clinical cut-off. Additionally, 23.8% of the mothers reported clinically relevant PTSD symptoms for their children.

### Association between caregiver and child report on PTSD symptoms

In our sample there was no significant correlation between the mean CRIES-13 total score and the children’s overall PTSD symptoms rated by the caregivers (*r* = .02, *p* = .85). Neither did we find a correlation between children’s and proxy-rated symptoms for intrusion, avoidance, or arousal. For details, see Table 1.

**Table 1:** Pearson correlation coefficient between CRIES-13 and PRCRS scores, including subscales

		*PRCRS* *total score*	*PRCRS* *intrusion*	*PRCRS* *avoidance*	*PRCRS* *arousal*
*CRIES-13 total score*	*r*	**.02**	−.03	.08	.06
	*p*	**.85**	.72	.30	.45
*CRIES-13 intrusion*	*r*	−.06	**−.11**	.001	−.05
	*p*	.42	**.15**	.99	.56
*CRIES-13 avoidance*	*r*	.03	.01	**.07**	.06
	*p*	.74	.91	**.36**	.42
*CRIES-13 arousal*	*r*	.07	.04	.10	**.11**
	*p*	.39	.60	.20	**.17**

*r = Pearson correlation coefficient; according subscales printed bold*

### The influence of socio-demographic variables on children’s PTSD self-ratings

To evaluate the influence of the children’s age, gender and living situation on their self-reported PTSD symptoms, multiple regression analyses were calculated. Neither age, nor gender nor the living situation had a significant influence on the CRIES-13 total score. Similar results were found for all three CRIES-13 subscales (see Table 2).

**Table 2:** Linear regression analyses: the influence of socio-demographic factors on self-reported PTSD symptoms of the children

		B	Std. Error	Standardized coefficient β	t	p-value	95% CI for B	
	Dependent variable						lower	upper
*CRIES-13 total score*	Age	0.14	0.32	0.03	0.43	0.67	− 0.49	0.76
	Sex	0.63	1.62	0.03	0.39	0.70	−2.57	3.83
	Living situation^a^	−1.57	1.95	−0.06	−0.80	0.42	−5.42	2.29
*CRIES-13* *intrusion*	Age	0.00	0.14	0.00	0.02	0.99	−0.28	0.28
	Sex	0.66	0.73	0.07	0.91	0.37	−0.78	2.09
	Living situation^a^	−0.39	0.87	−0.04	− 0.45	0.66	−2.12	1.34
*CRIES-13* *avoidance*	Age	0.03	0.16	0.02	0.19	0.85	−0.29	0.35
	Sex	0.65	0.82	0.06	0.79	0.43	−0.98	2.28
	Living situation^a^	0.45	0.99	0.04	0.46	0.65	−1.51	2.41
*CRIES-13 arousal*	Age	0.10	0.14	0.06	0.73	0.47	−0.17	0.38
	Sex	− 0.68	0.72	− 0.07	−0.94	0.35	−2.10	0.75
	Living situation^a^	−1.63	0.87	−0.15	−1.88	0.06	−3.34	0.09

^*a*^ *dichotomized variable: 0 = village, 1 = SOS village; CI = confidence interval*

### Mothers’ trauma symptoms and ratings of children’s trauma symptoms

In our sample, there was a highly significant positive association between the mother’s own PTSD-symptom load and their rating of the child’s PTSD symptoms (*β* = .52, *p* < .001), even after controlling for the child’s age, gender, self-reported PTSD symptoms or living situation. The model explained 36.0% of the variance of the PRCRS total score.

This effect was also found for the three trauma-related subscales of the PRCRS: highest associations with the mothers PTSD-symptoms were found for avoidance behaviour (*β* = .31, *p* < .001) and arousal (*β* = .30, p < .001). The association with intrusion was slightly smaller, yet still significant (*β* = .19, *p* = .04). Together these results highly indicate that mothers, who had more self-reported trauma symptoms, rated their children as more symptomatic.

### Factors influencing mother-child discrepancies in the rating of children’s PTSD

Overall, mothers reported scores above the clinical cut-off for their children significantly more often than children themselves (23.8% vs. 12.8%; *χ*^2^ = 6.61, *p* = .010). To evaluate which factors influence the ‘correct’ assessment of both parties (i.e. corresponding ratings), a logistic regression analysis was conducted. Children’s age, sex, living situation as well as the mother’s PTSD symptoms were entered as independent variables. In this multivariate analysis, the mother’s PTSD symptoms were the only significant predictor for discrepancies in the rating of the child’s PTSD: if mothers reported clinically relevant PTSD-symptoms, the likelihood for disagreement on the child’s PTSD ratings more than doubled (see Table 3).

**Table 3:** Multivariate logistic regression model: Influential factors on mother-child accordance regarding children’s PTSD

	Regression coefficient B	Wald	df	Sig.	Adjusted OR	95% Confidence Interval for OR	
						Lower	Upper
Age	−0.09	1.71	1	.19	0.91	0.80	1.05
Sex	0.37	1.08	1	.30	1.44	0.72	2.89
Living situation	0.08	0.03	1	.85	1.08	0.46	2.53
PTSD mother (0 = PTSD; 1 = no PTSD)	0.85	5.49	1	.019	2.34	1.15	4.76

*OR = Odds Ratio*

## Discussion

The main objective of the current study was to examine the agreement of Tsunami-affected mothers and children for posttraumatic stress symptoms in a non-Western – South Indian – sample four years post-disaster. In addition to the child-mother agreement on trauma symptoms, children’s age, gender, living situation and the mothers’ own trauma symptomatology on this concordance was investigated. Children’s self-ratings on their trauma symptoms were also examined with regard to their age, gender and living situation. In general, we found similar findings to Western cultures, i.e. that caregiver-child agreement on posttraumatic stress reactions was poor [6]. Moreover, mothers’ own trauma symptoms played a large role in their assessment of the children’s symptoms.

In line with our expectation was the missing significant correlation between mothers’ external assessment of the total PTSD symptom score as well as the three PTSD symptom clusters and children’s self-reported total PTSD symptom score and respective PTSD criterions. On the one hand, this finding is not surprising and in full agreement with a majority of scholars. For example, Jensen et al. [11], who examined 1.285 parent-child dyads with regard to five major diagnostic categories (anxiety, depression/dysthymia, Attention Deficit Hyperactivity Disorder/ADHD, Oppositional Defiant Disorder/ODD, and conduct disorder), found that the child-caregiver concordance was poor, irrespective of the type of diagnosis. On the other hand, there are numerous studies that prove a generally higher agreement between caregivers and children when they rate observable/external domains compared to un-observable/internal domains [10, 14]. This tendency transferred to the PTSD symptomatology means that the parent-child agreement should be higher for the observable hyper-arousal criterion than for the internal intrusion and avoidance criterions [17], but in the present study no improvement on the mother-child rating could be found for the hyper-arousal cluster. However, the very weak associations were somewhat surprising when viewed against the highly collectivistic socio-cultural background of our sample. It is assumed that parent-child discrepancies may be smaller in societies where cultural values promote familism and collectivism [45] than in societies that promote individualism and autonomy. Familism emphasises prioritising the family over the individual, showing respect for elders, and honouring the family name, thus familism reflects a collectivist value system [45]. The findings of the study of Rescorla et al. [46] on parent-adolescent agreement in 25 societies slightly indicated that familism might explain some of the differences because such cultures have strong norms and low tolerance of deviant behaviour [47].

In contrast to several scholars who reported that demographic characteristics of children such as age and gender have a positive influence on caregiver-child concordance for different diagnostic classifications (age: [19, 20, 48] gender: [13]), our findings clearly showed that children’s age and gender did not account in any way for the discrepancies in mother versus child reports. This result did not correspond to our hypothesis, nevertheless it is not contradictory to the scholarly literature. Study findings on this topic are inconsistent and show insignificant relations between child demographic characteristics and caregiver-child agreement [12, 21]. In addition, we found that a child’s living situation had no influence on mother-child discrepancies for PTSD ratings. However, research on caregiver-child agreement specifically for PTSD is scarce and shows no clear indication whether a child’s age and gender are related to informant discrepancies or not [18, 24]. Some researchers suggest that parent-child agreement is less influenced by children’s age and gender than by quantitative (the time children and caregivers spend together) and qualitative (e.g., acceptance, family conflicts) aspects of parent-child relationships [49]. Discrepancies in parent-child reports have been related to high ratings in family conflicts [13], low parental warmth and acceptance, and less spending time together [49]. Even though we did not collect data on quantitative and qualitative aspects of mother-child relationships, the little time mothers and children spent together could be an explanation for the missing concordance in our study. For example, Achenbach [50] noted that parent-child discrepancies might arise due to parents’ inability to observe their children where they are not present. In the qualitative questioning of Exenberger and Juen [33], mothers complained heavily about their work overload. They had extremely long working hours in the fishing business and consequently little time for their children [33].

Against our hypothesis, children of the current study did not show any age and gender differences of self-reported trauma symptoms. In general, studies have shown contradictory results concerning age and gender differences in child self-reported PTSD symptoms. Some studies tended to find that girls report more trauma symptoms than boys [25], whereas other work did not find such an effect [26, 27, 29]. With regard to age there are also mixed findings where some authors detected no age differences [28, 29], and others reported that older children were more symptomatic [51]. However, the results of our study were surprising, especially regarding gender, given the fact that in India female gender determines lower status because there is a clear preference for sons over daughters [52]. Even though this kind of fertility pattern – daughter aversion – has declined in the last two decades in Tamil Nadu (where the present study took place) having a son yet guarantees that no large dowries in marriages need to be provided [53]. Thus, sons are a less economic burden for the family than daughters [52, 53]. There was also no support for our hypothesis that children who lived with their parent(s) in a fishing village did not show higher self-rated trauma symptoms than children living in a SOS Children’s Village. This was a surprising result because in the focus groups of Exenberger and Juen’s study [33], both mothers and children living in fishing villages emphasised that reminders of the Tsunami (e.g., full moon, higher water level) triggered children’s fear even four years post-event, whereas SOS children were less exposed to these triggers.

In line with our hypothesis was the finding that mothers’ own trauma symptoms had a significant impact on their child ratings. Symptomatic mothers reported significantly more avoidance, intrusion and hyper-arousal in their children. Moreover, symptomatic mothers even rated more symptoms for their children compared to the children’s self-ratings as also shown in the study of Kassam-Adams et al. [32]. Mothers’ estimations of trauma symptoms in the child, therefore, may be highly influenced by their own subjective traumatic experience and PTSD symptoms. Mothers’ PTSD symptoms were even the only significant predictor for discrepancies in the rating of child PTSD in our study. Consequently, our results also might provide an insight in a mother’s own level of posttraumatic stress. The findings of the above mentioned qualitative questioning of Exenberger and Juen [33] indicated that mothers tell something about their own mental health status when they rated posttraumatic stress in their children. These results resemble those of Shemesh et al. [24] who found that parents’ own PTSD symptoms were associated with their reports of their child’s symptoms regardless of whether the parents experienced the same traumatic event as the child or not. Furthermore, the authors pointed out that parents’ reports of child PTSD symptoms offer insights in parents’ own level of posttraumatic stress.

The main limitation of this study includes the fact that different assessment instruments for the estimation of child PTSD symptoms were used. Mothers’ external assessment of children’s PTSD symptoms was based on the PRCRS [41], and children’s self-report on their trauma symptoms were grounded on the CRIES-13 [38]. It would have been preferable to use the same assessment instruments in terms of a child and parent version so that mothers and children’s estimations of trauma symptoms could have been compared. Another limitation is that no explicit data have been collected on the mother-child relations, because more information about their everyday family life might have provided more accurate explanations for the missing mother-child concordance. We have only indirect information about this relationship because of the mothers’ unintentional information they revealed in the qualitative questioning of Exenberger and Juen [33].

## Conclusion

Nevertheless, in conclusion, this study underscores the importance of assessing mothers’ own posttraumatic stress reactions when estimating children’s PTSD symptomatology because discrepancies in mother-child ratings seem to be linked to mothers’ own long-term trauma symptoms. The missing concordance between mothers and children on children’s trauma symptoms did not reflect cultural characteristics. It is rather an indication that after the Tsunami a structured everyday life could not be established, which would have been necessary for a good relationship between children and caregivers. Based on the assumption that the relationship quality between child and caregiver moderates child-caregiver discrepancies on child trauma symptoms [49], the focus of inquiry in the long- as well as in the short-term aftermath of disaster should be on parent-child relationships. This information could help to get more insight in the interplay of life circumstances and external assessment of trauma symptoms in the long-term aftermath of disasters. Consequently, future research comparing informants’ perceptions of family functioning should be the focus of parent-child agreement because family functioning is assumed to explain cross-informant discrepancies in different cultures [54].

## Data Availability

Data is not available for online access, however readers who wish to gain access to the data can write to the to the first author Silvia Exenberger at silvia.exenberger-vanham@i-med.ac.at with their requests, which would be subject to ethical approval.

## Abbreviations

ADHD: Attention Deficit Hyperactivity Disorder
ASD: Acute Stress Disorder
CRIES-13: Children’s Revised Impact of Event Scale-13
DSM: Diagnostic and Statistical Manual of Mental Disorders
IES-R: Impact of Event Scale-Revised
NGO: non-governmental organisation
ODD: Oppositional Defiant Disorder
PRCRS: Parent Report of the Child’s Reaction to Stress
PTS: Posttraumatic Stress Symptoms
PTSD: Posttraumatic Stress Disorder
